# Googling Boundaries for Operating Mobile Stroke Unit for Stroke Codes

**DOI:** 10.3389/fneur.2019.00331

**Published:** 2019-04-04

**Authors:** Thanh G. Phan, Richard Beare, Mark Parsons, Henry Zhao, Stephen Davis, Geoffrey A. Donnan, Velandai Srikanth, Henry Ma

**Affiliations:** ^1^Stroke Unit, Clinical Trials Imaging and Informatics Division of Stroke and Aging Research Group, Monash Medical Centre, Monash University, Clayton, VIC, Australia; ^2^Department of Medicine, Peninsula Health, Melbourne University, Frankston, VIC, Australia; ^3^Melbourne Brain Centre, Melbourne University, Parkville, VIC, Australia; ^4^Florey Neuroscience Institute, Melbourne University, Parkville, VIC, Australia

**Keywords:** stroke, transport, modeling, optimization, Google Map

## Abstract

**Background:** Mobile stroke units (MSU) have been proposed to expedite delivery of recombinant tissue plasminogen activator (tPA) and expedite endovascular clot retrieval (ECR). Unexplored questions in the use of MSU include: maximal distance from base, time limit with regards to the use CT imaging, CT Angiography, CT Perfusion, and Telemedicine. We developed a computational model as an app (https://gntem3.shinyapps.io/ambmc/), taking into account traveling time to explore this issue. The aim of this study was to define the operating parameters for an MSU in a large metropolitan city, based on the geography of Melbourne.

**Methods:** There are 2 hospitals (Royal Melbourne Hospital/RMH, Monash Medical Center/MMC) designated to provide state-wide ECR services. In these spatial simulations, the MSU is based at RMH and delivers tPA at the patient's pick-up address and then takes the patient to the nearest ECR center. We extracted the geocode of suburbs in Melbourne and travel time to each hospital using *ggmap*, an interface to Google Map API. The app contains widgets for varying the processing time at the patient location (default = 30 min), performing CT angiography (default = 10 min), performing telemedicine consultation (default = 15 min). The data were compared against those for usual ambulance metrics (default traveling time = 15 min, processing time at patient's location = 20 min, door to tPA = 60 min, door to groin = 90 min). Varying the widgets allow the viewer to explore the trade-off between the variable of interest and time to therapy at a suburb level.

**Results:** The MSU was superior for delivering tPA to all Melbourne suburbs (up to 76 min from RMH). If the CTA times or processing time at location increased by 20 min then it was superior for providing ECR to only 74.9% of suburbs if the return base was RMH. Addition of CT Perfusion or telemedicine consultation affect the ability of a single hospital to provide ECR but not tPA if these additions can be limited to 20 min. Conclusion: The app can help to define how best to deploy the MSU across Melbourne. This app can be modified and used to optimize operating characteristics of MSU in other centers around the world.

## Introduction

Stroke is a leading cause of disability worldwide and results in significant economic and societal cost (1). In spite of this, there is now substantial optimism with acute stroke management since the publication of pivotal trials for thrombolysis (2) and endovascular clot retrieval (ECR) (3–8). Substantial efforts go into providing thrombolytic therapy within the “golden hour.” With this in mind several groups around the world have used mobile stroke unit or ambulance equipped with CT scanner to achieve this aim. The first randomized trial showed an absolute difference of 16 min from onset to treatment with TPA (9).

Several models of mobile stroke unit (MSU) are currently in existence. The Berlin model operates an MSU within 16 min radius from base (10). However, other models have not defined a clear distance from the treating hospital (11). It is not clear if the distance/time threshold described in the Berlin model also applies to Australia (or elsewhere). Further, the original MSU model was described in an ambulance performing only non-contrast CT (NCCT) (10) with the aim of providing faster tPA delivery. Since then, CT angiography (CTA) and Telemedicine have been added to MSU (2). However, it is not clear if CT Perfusion is currently in use on MSU (11). These latter additions permit exploration of triaging delivery of patients with large vessel occlusion to ECR-capable hospitals. Unexplored questions in the use of MSU include, maximal travel time from base, limit with regards to the time spent on use of additional CT imaging (CTA, CTP), and time spent using Telemedicine on board the MSU instead of having a stroke doctor on board. We propose the use of Google Map API for transport modeling of MSU in metropolitan cities in Australia (12).

The state of Victoria, Australia has set up a statewide service protocol and two hospitals designated as ECR hubs (Royal Melbourne Hospital/RMH and Monash Medical Center/MMC). The purchase cost of MSU has been estimated to be around US$ 750,000 to US$1,400,00 with annual running cost of around US$1,000,000 (13). As such, only one model is operating in Melbourne since 2017. By taking advantage of recent developments in the Google Map application program interface (API), we undertook this simulation study to develop and apply a computational method to objectively define the operating parameters for MSU in Melbourne from alarm to reperfusion therapy time.

## Methods

### Setting

Melbourne is the capital city of the state of Victoria in Australia with a population of ~4.9 million and population density of 12,400 per km^2^ (http://www.abs.gov.au/websitedbs/). The postcodes for metropolitan Melbourne are in the range 3000–3207. These ECR hubs are required to provide a 24-h service not just for patients in their immediate local catchment but also for all residents of Victoria. These catchment of the ECR hubs is displayed at https://gntem2.github.io/Google-Map-to-Victorian-ECR-Hospitals/. In addition to these 2 centers, there are 2 further ECR capable hospitals and 6 non-ECR capable hospitals providing intravenous thrombolysis in metropolitan Melbourne.

### Google Map API

We used *ggmap* to access Google Map application program interface (API) (https://developers.google.com/maps/). Ggmap is an open source software freely available written in R (R Project for Statistical Computing, version 3.4.4) (14). The Google Map “geocoding” API describes a location in terms of its geocode (latitude and longitude). We performed geocoding of the centroid of each postcode. The traveling time to and from each hospital in Melbourne was obtained from Google Map “distance matrix” API. We found the Google Map “distance matrix” API to be faster than the Google Map “direction” API; this is due to the latter also obtaining a direction matrix for each query.

### Experiments

Currently, MSU operates within a 20 km radius from RMH. The spatial simulations were performed without restriction on operating distance. The MSU is assumed to be based at RMH and delivers TPA at the patient's location and returns to base (RMH) for ECR. In the second scenario, the MSU delivers patient to both RMH and MMC for ECR.

### Optimization

We set up one set of linear equations for MSU and one for usual ambulance. The total sum of time taken for MSU and usual ambulance were compared to obtain the number of postcodes where one model is superior to the other in terms of time from alarm to reperfusion therapy time. The simulation for tPA was performed with default time to therapy for usual ambulance delivery (from pick up) set at 95 min and for ECR at 125 min. Due to the multiple ambulance hubs around Melbourne, we made the assumption for usual ambulance transport is that the patient is 15 min from the nearest ambulance, and that on arrival it takes 20 min to process the patient at the scene. These data were based on the validation data between observed ambulance time and Google Map API queries in our previous publication (12). In this study, it is assumed that each hospital can give tPA within 60 min of hospital arrival; this time include performing advanced imaging with CT Perfusion and CT Angiography (15). By contrast, MSU take a certain time to arrive at patient location, takes another 30 min to process patient at scene (including blood test). The CTA is assigned default time of 10 min to set up (inclusive of setting up contrast media injector and processing of images). It is assumed that once large vessel occlusion is identified the patient is taken to ECR hub for clot retrieval and that there is no need to the ECR hub to do further imaging. A processing time (default 30 min) at the hub is assigned for viewing CTA images and setting up ECR procedure.

The app contains widgets for varying the processing time at the patient location and performing non-contrast CT/NCCT (default = 30 min), performing CT angiography (default = 10 min), performing telemedicine consultation (default = 15 min) (16). The data were compared against those for usual ambulance metrics (default traveling time = 15 min, processing time at patient's location = 20 min, door to TPA = 60 min, door to groin = 90 min). Varying the widgets allow the viewer to explore the trade-off between the variable of interest and time to therapy at a post code level. The reader can choose to change these variables by going to the app. Internet connectivity is required for app access.

### Web Based Display

The interactive web-based maps were generated using R package *leaflet* using tiles from OpenStreetMap (OpenStreetMap contributors. For copyright see www.openstreetmap.org/copyright) (17). Next, these web pages were incorporated into app, written using Shiny, Rstudio, and is accessible at https://gntem3.shinyapps.io/ambmc/.

## Results

**Figure 1:**
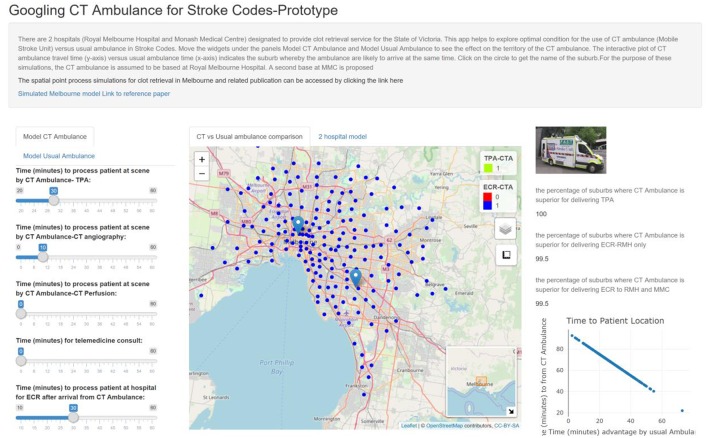
The apps contain widgets (on the left side) for varying variables of interest for MSU and usual ambulance. The suburbs in which MSU is superior to usual ambulance is displayed on the map as green (inferior as yellow). The viewer can switch the control box to display suburbs in which MSU is superior for returning to base at RMH to perform ECR (blue). The percentage of suburbs in which MSU is superior to usual ambulance is displayed on the right-hand side. Using CTA & on-board neurologist, MSU can reach 100% of suburbs for TPA.

Using the default setting for tPA [MSU = 40 min (processing at scene = 30 min, CTA = 10 min)], MSU is superior to usual ambulance (95 min) in terms of time metric for delivering tPA to 100% of metropolitan Melbourne suburbs (see Table 1, Figure 1). Delivery of tPA in Melbourne can be done faster by tPA as far as 76 min from the base at RMH. Provision of ECR was superior to usual ambulance route in terms of time metric to 99.5% of suburbs if the patients are brought back to RMH for therapy. If MSU also uses MMC as a site for ECR then this model is superior in 99.5% of suburbs.

**Table 1:** Effect of varying time MSU metrics on performance.

**Usual ambulance to TPA/ECR (min)**	**MSU**						
	**Processing time (min)**	**CTA (min)**	**CTP (min)**	**Telemedicine (min)**	**Superior for TPA (%)**	**Superior for ECR (RMH) (%)**	**Superior for ECR (RMH and MMC) (%)**
95/125	30	10			100	99.5	99.5
95/125	30	10	10		99.5	92.3	98.4
95/125	30	10		10	99.5	92.3	98.4
95/125	30	10	10	10	97.8	74.9	93.4
95/125	30	10		20	97.8	74.9	93.4
95/125	30	10		30	93.4	39.3	63.9
85/115	30	10			99.5	92.3	98.4
85/115	30	10		10	97.8	74.9	93.4
85/115	30	10		20	93.4	39.3	63.9
75/105	30	10			97.8	74.9	93.4
75/105	30	10		10	93.4	39.3	63.9
75/105	30	10		20	60.1	13.7	21.9

### Improved Door to tTPA/Groin

If the delivery of therapy by usual ambulance route is faster by 10 min (by reducing door to tPA) then MSU is superior for ECR in 92.3% of suburbs when treatment is performed at base at RMH and 98.4% of suburbs when treatment can be performed at RMH and MMC (see Table 1). If the delivery of therapy by usual ambulance route is faster by 20 min, then MSU is superior for ECR by time metric in 74.9% of suburbs (treat at base) and 93.4% of suburbs (treat at base and MMC).

### Addition of CT Perfusion to Imaging Protocol

Using time metric, MSU remained superior to usual ambulance for TPA in 99.5% of cases when CT Perfusion was added to the imaging protocol. Provision of ECR is superior to usual ambulance in 92.3% of suburbs if the patients are brought back to RMH for therapy. If MSU also uses MMC as site for therapy then this model is superior in 98.4% of suburbs (Table 1).

### Addition of Telemedicine

The results for telemedicine was similar to that described above for CT Perfusion. Using time metric, MSU remained superior to usual ambulance for tPA in 99.5% of case when Telemedicine adds 10 min to the treatment time. When Telemedicine adds 20 min to the treatment time MSU remained superior to usual ambulance for TPA in 97.8% of suburbs and for ECR in 74.9% of suburbs when returning to base (RMH only) and 93.4% of suburbs when returning to either ECR hubs (RMH and MMC). When Telemedicine adds 30 min to the treatment time MSU remained superior to usual ambulance route for TPA in 93.4% of suburbs and for ECR in 39.3% of suburbs when returning to base (RMH only) and 63.9% of suburbs when returning to either ECR hubs (RMH and MMC).

### Combined CT Perfusion and Telemedicine

Using time metric, MSU remained superior to usual ambulance for TPA in 96.2% of case when CT Perfusion was added to the imaging protocol. Provision of ECR is superior to usual ambulance in 58.5% of suburbs if the patients are brought back to RMH for therapy. If MSU also uses MMC as site for therapy then this model is superior in 83.1% of suburbs.

## Discussion

In this study, we described an objective method to explore operating characteristics of a MSU using time to therapy as metric of performance. Further, the time metric is displayed on a map of Melbourne, permitting optimization of the operating parameters at spatial level. The concept behind simulation to define operating characteristics of the MSU is that one cannot know prior to deployment the operating parameters of MSU. These parameters affect the choice of MSU e.g., addition of CT Angiography and CT Perfusion. Purchase of these additional equipment is expensive but may also negatively prolong the time to treatment. Further should the neurologist be on board or should it be staffed by trained nurse supported by telemedicine? The app can be used strategically for planning. Exploration of the app shows that MSU can be deployed across Melbourne (maximum range of 76 min for TPA). At this range and employing time metric for assessment, MSU is still superior for delivery of ECR if the 2 hubs model is used. The modeling approach described here can be used to define boundaries for operating MSU around the world.

The hypothetical range of MSU in our studies (76 min) is much further that used in Berlin (16 min, population 1.3 million) (10). It is not clear the reason for the short operating range in Berlin. However, operating MSU at long range may decrease capability to respond to another case especially in Melbourne which has a large catchment (~4.9 million). Other models have not described the operating range but have described the population and which may serve as proxy for the catchment. The New Jersey model serves a modest population of 460,000 (13) and the Cleveland model serves a population of 390,000 (18). There are attempts to explore the use of MSU in rural setting in Germany (11). We use the app to propose the use of spatial simulation to help define the operating parameters of MSU.

**Figure 2:**
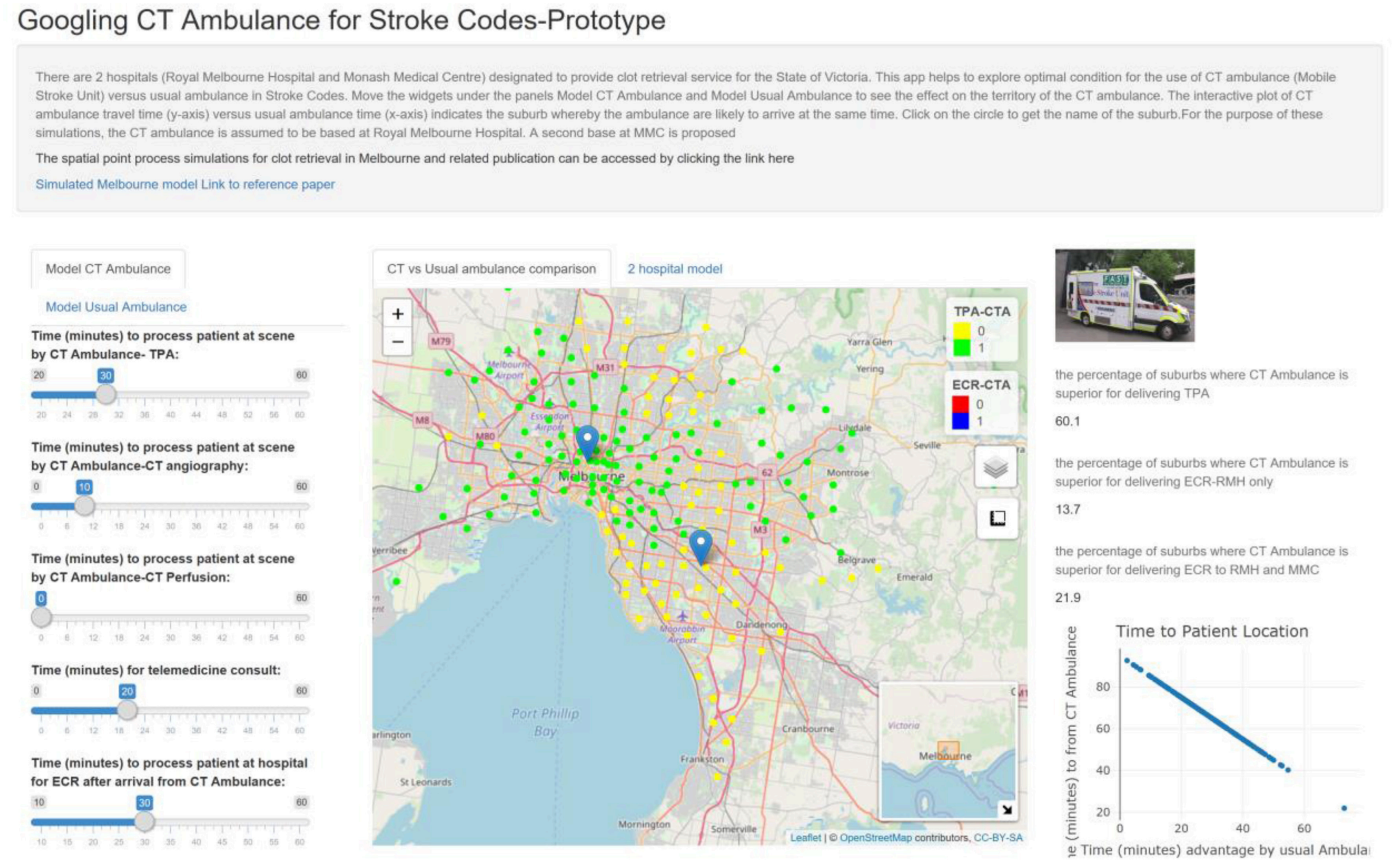
The apps contain widgets (on the left side) for varying variables of interest for MSU and usual ambulance. The suburbs in which MSU is superior to usual ambulance is displayed on the map as green (inferior as yellow). The viewer can switch the control box to display suburbs in which MSU is superior for returning to base at RMH to perform ECR (blue). The percentage of suburbs in which MSU is superior to usual ambulance is displayed on the right-hand side. If the hospital can reduce door to TPA from 60 to 40 min by usual ambulance route, then MSU can reach 60.1% of metropolitan Melbourne for TPA.

The earlier studies described the use of MSU with NCCT capability only (10, 13, 16). As such the data regarding performance with CTA and CTP are not well known (11). Our study shows that the addition of further test such as CTP comes at the expense of reducing the operating distance for MSU. The idea behind using CTP to triage patients for tPA and ECR with go no go maps (salvageable tissue vs. large infarct core) (7, 19, 20). In this simulation, CTP did not confer a benefit as the metric of comparison with usual ambulance delivery was time based; further, this metric assumes that decisions are correctly made with CTA. In this study, the default time for CTP was 10 min. This time included reloading the contrast media injector, acquire the scan and process the perfusion image. This time frame is what we have observed in a clinical scanner in our hospital. In the future, newer scanner may combine CTA and CTP into one image acquisition rather than 2 acquisitions at present. Additionally, the acquisition and processing time may reduce significantly. The viewer can explore this by moving the widget to a time below the default 10 min. In these future scenarios with improved scanner, it would be advantageous to perform CTA and CTP. The impact of these new technology on running MSU with Telemedicine will be expanded below. In these simulations we had empirically set door to needle time at 60 min (including time for performing advanced imaging studies such as CT Perfusion and CT Angiography). We do not have access to individual site's door to needle time metric. We have provided a widget under the tab Usual Ambulance so that the user can explore the data. The data shows that when the door to needle time is 45 min by usual ambulance route, MSU is superior in 99.5% of suburbs for tPA, 83.1% of suburbs for ECR at base and 97.3% of suburbs for ECR at base and MMC (Figure 2).

The use of telemedicine in MSU has been proposed in several studies (13, 18, 21). In a direct comparison between usual ambulance and MSU with telemedicine, the latter method results in earlier CT scan time (by 23 min) and TPA (by 39 min) (18). These studies have used scenarios played by actors and compare performance between on board neurologist and tele-neurologist (21). In these actor scenarios, there was no difference in time to TPA between on-board and tele-neurologists. Technical failure was reported in 2% of study employing actors for simulation (16) and deemed to be significant in 2% with “live” cases described by New Jersey group (13). In that study, **internet** reconnection was required in 39% (35/89) of cases. The Cleveland groups (18) did not described technical failure with telemedicine. Treatment of stroke mimics with telemedicine was estimated at ~20% (13). This rate is comparable with the range of stroke mimics presenting to hospital. However, the time metric used in our study cannot evaluate this aspect.

Provision of telemedicine with modern CT Angiography or CT Perfusion add further complexity with transmission of data, not evaluated in earlier reports (13, 18, 21). We had optimistically set 15 min as the default Telemedicine consultation time and which include transfer of images from MSU back to base. In the absence of the on-board neurologist, transfer of images back to base is required both for clinical reading and clinical governance. Further, the default setting here for CT Perfusion is optimistic as there is time cost for processing of perfusion images for determining presence of salvageable tissue.

In this study, we have used a novel and objective computational method to map the service boundaries for MSU based on travel time to the hub. This work was based on similar strategies for mapping ECR boundary in Melbourne and Adelaide (12). The works performed here used software packages (*ggmap* and *leaflet)* within the R environment, due to our familiarity with these packages. These are not the only R packages that can used for geospatial works and interactive display of map. Other R packages for geospatial analysis include *googleway* (access Google Map API) and *taRifx.geo* (access Google and Bing Map API) (22)*;* and for map display, there is *plotGoogleMaps* package. Outside of R environment, investigators can use python (23), matlab (24), javascript, and java.

Our study has several limitations outline below. It can be criticized as giving unfair advantage to the MSU as most of the simulations in MSU were performed without CT Perfusion. By contrast, the Usual Ambulance has a CT Perfusion arm which add 10 min to the hospital processing time. When this 10 min advantage has been removed, the MSU approach is still superior for delivery of TPA in 99.5% of suburbs, provision of ECR at RMH base in 92.3% of suburbs and provision of ECR at RMH or MMC in 98.4% of suburbs. The reader can click on the tab Model for Usual Ambulance and adjust the widget “Time (min) to process patient at hospital for TPA after arrival-usual ambulance.” The app can be construed as simplistic in its focus on superiority of one model over another in terms of alarm to reperfusion therapy time and not focus on other aspect decision on reperfusion such as clinical variables, LVO status and angiographic suite availability. In this study we had made the assumption that availability of angiographic suite is high given that designated ECR hub in Melbourne are required to have two angiographic suites. This use of the app is different from that used by others for field triaging of stroke (25). We had performed the modeling with RMH as base of MSU for strategic planning and modeling. We did not analyze the possibilities of MSU being at different locations around Melbourne. With the 76 min radius, the MSU can cover metropolitan Melbourne easily and respond to call on the West side of Melbourne after delivering care in the East side. We had explored this possibility by interrogating Google Map API during peak hour traffic and found the traveling time was within the 76 min.

The app was developed focused on time metric for defining operating characteristics of MSU from alarm to reperfusion therapy time. However, MSU has other benefit including its use in field triaging of hospital destination for definitive therapy when the MSU is combined with CTA. In this role it can be combined with non-MSU ambulance to take the patient to the destination and the MSU can proceed to the next stroke code. This role of MSU for field triaging of therapy cannot easily be provided by using CT only. Finally, the app was intended for strategic decision making about the operating characteristic of the MSU. It can be modified for day to day analysis if needed. We have provided the source code on github account for anyone wishing to make changes (https://github.com/GNtem2/AmbMC).

In this study we proposed that the travel time from Google Map API were similar to that observed when traveling on ambulance. In our previous analysis, we had found this the difference between the travel time is in order of min (12). One drawback of this study is that it assumes that time cost is the main consideration when deciding hardware and software (CTA and CTP) or method of stroke diagnosis (on board neurologist or telemedicine). An earlier study had used data from meta-analysis of TPA to project cost benefit ratio with MSU (26). Other study described cost relating to purchase and maintenance of connectivity devices for telemedicine (13). There are current attempts data for cost effectiveness analysis and hopefully these papers will be available in the near future (11). An additional variable is sustainability of the work force and which depends on the number of stroke (vascular) neurologist available work on-board MSU (27).

In summary, we have provided a data driven approach to determine the operating parameters as well as location of base of MSU. Our recommendation is that MSU can be performed with CT Angiography, if the intention is to use MSU to triage site for ECR. The use of CT Perfusion and Telemedicine require further consideration and planning. This recommendation would change in the future as technology improves and acquisition and processing time can be significantly reduced. This approach can be used where Google Map API is available.

## Data Availability

The datasets generated for this study are available on request to the corresponding author.

## Author Contributions

TP and HM: design and concept; TP and RB: coding and data analysis; TP, RB, HM, VS, MP, HZ, SD, and GD: writing.

### Conflict of Interest Statement

The authors declare that the research was conducted in the absence of any commercial or financial relationships that could be construed as a potential conflict of interest.
